# Beetle iridescence induces an avoidance response in naïve avian predators

**DOI:** 10.1016/j.anbehav.2022.04.005

**Published:** 2022-06

**Authors:** Karin Kjernsmo, Anna M. Lim, Rox Middleton, Joanna R. Hall, Leah M. Costello, Heather M. Whitney, Nicholas E. Scott-Samuel, Innes C. Cuthill

**Affiliations:** a School of Biological Sciences, University of Bristol, Bristol, U.K; b School of Psychological Science, University of Bristol, Bristol, U.K

**Keywords:** aposematism, *Gallus gallus domesticus*, gloss, iridescence, predation, protective coloration, specular reflection, *Sternocera aequisignata*

## Abstract

It has recently been found that iridescence, a taxonomically widespread form of animal coloration defined by a change in hue with viewing angle, can act as a highly effective form of camouflage. However, little is known about whether iridescence can confer a survival benefit to prey postdetection and, if so, which optical properties of iridescent prey are important for this putative protective function. Here, we tested the effects of both iridescence and surface gloss (i.e. specular reflection) on the attack behaviour of prey-naïve avian predators. Using real and artificial jewel beetle, *Sternocera aequisignata*, wing cases, we found that iridescence provides initial protection against avian predation by significantly reducing the willingness to attack. Importantly, we found that the main factor explaining this aversion is iridescence, not multiple colours per se, with surface gloss also having an independent effect. Our results are important because they demonstrate that even when prey are presented up close and against a mismatching background, iridescence may confer a survival benefit by inducing hesitation or even, as sometimes observed, an aversion response in attacking birds. Furthermore, this means that even postdetection, prey do not necessarily need to have secondary defences such as sharp spines or toxins for iridescence to have a protective effect. Taken together, our results suggest that reduced avian predation could facilitate the initial evolution of iridescence in many species of insects and that it is the defining feature of iridescence, its colour changeability, that is important for this effect.

## Highlights

• Iridescence and gloss are usually associated with display, but can they also defend?
• We tested naïve birds' willingness to attack glossy and iridescent prey.
• Iridescence reduced attack probability, with gloss also having an independent effect.
• Iridescence and gloss could provide a survival benefit to prey, even postdetection.

Iridescence is a striking form of structural coloration in which hue and intensity vary with the angle of view or illumination (Doucet & Meadows, 2009; Moyroud et al., 2017; Stavenga et al., 2011). Our understanding of the optical properties of iridescence has increased significantly since initial descriptions by Robert Hooke (1665), but our understanding of its biological function, beyond sexual selection, is limited (Doucet & Meadows, 2009). Only recently has empirical support for an antipredator function of iridescence started to accumulate, providing insights as to why this form of coloration has evolved independently in many sexually monomorphic species and even nonreproductive stages. Two common forms of iridescence, those produced by diffraction gratings or multilayers, impair shape recognition by insects (Kjernsmo et al., 2018) and multilayer cuticle reflectors of jewel beetles reduce detectability by birds and humans (Kjernsmo et al., 2020). Impaired object detection and recognition are hallmarks of camouflage (Cuthill, 2019; Merilaita et al., 2017). Postdetection, Pike (2015) demonstrated that changing colours can also reduce the strike accuracy of predators.

Another putative antipredator function of iridescence is aposematism: signalling unprofitability (e.g. Fabricant et al., 2014; Schultz, 2001, Waldron et al., 2017). However, data for this are ambiguous. One recent study found no effect of iridescence on warning signal efficacy (Pegram et al., 2015). In a separate investigation of iridescence in chemically defended leaf beetles, *Oreina cacaliae*, Waldron et al. (2017) showed that an increase in specular reflection enhanced avoidance learning in birds. Specular reflection, associated with glossy surfaces, shares with iridescence a variation in intensity with angle of viewing, but the hue does not change (Franklin & Ospina-Rozo, 2021). As Waldron et al. (2017) manipulated only gloss, the potential effect of changes in hue, i.e. iridescence, is unknown.

One unexplored effect of iridescence, relevant to both the rate of avoidance learning and evolutionary considerations of initial viability, is whether it induces an aversion response in naïve predators. To investigate this, and to isolate the effects of gloss and iridescence, we measured the attack willingness of naïve chicks of the domestic fowl, *Gallus gallus domesticus*, presented with real and artificial jewel beetle, *Sternocera aequisignata*, wing cases, all of which were conspicuous on the background against which they were displayed. We used both iridescent and noniridescent prey with the same overall range of colours, as well as manipulating their level of specular reflection (gloss). We predicted that if iridescence (where hue and intensity vary with angle) is an important factor in predator aversion, the birds would be less willing to attack the iridescent than the noniridescent prey. Alternatively, if gloss (where just intensity varies with angle) is more influential, the birds would be less willing to attack the prey items with higher specularity.

## Methods

### Stimuli

**Figure 1:**
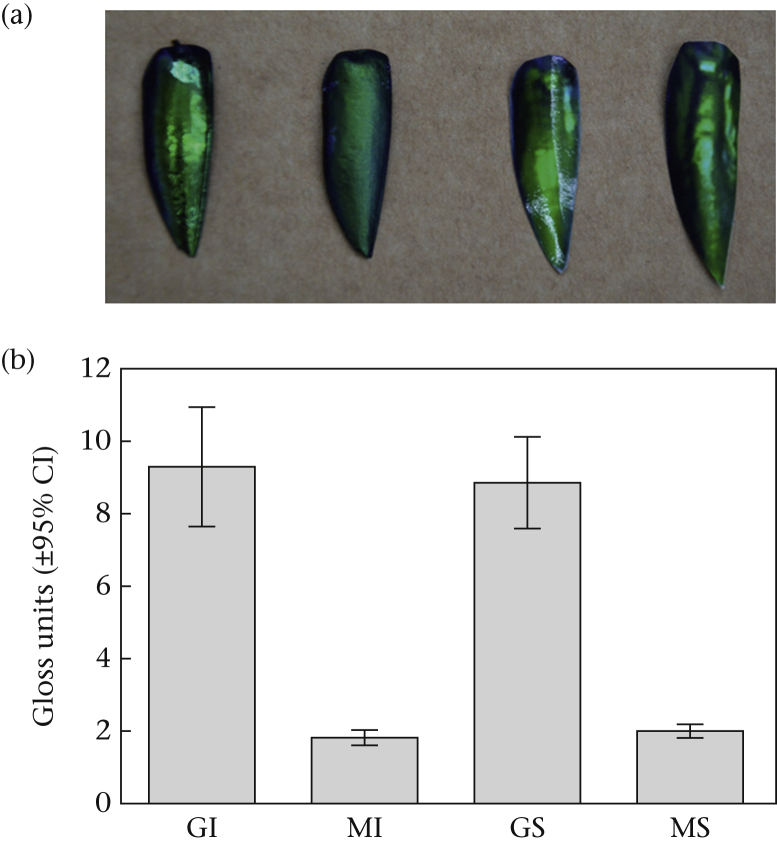
(a) The four different experimental stimuli photographed on the foraging plate. From left to right: glossy iridescent (GI), matte iridescent (MI), glossy static spectrum (GS) and matte static spectrum (MS). (b) Mean (± 95% CI) levels of gloss reflected for 10 randomly selected targets for each treatment group.

The experiment was a 2 × 2 factorial design: iridescent versus noniridescent, and gloss versus matte (Fig. 1a, Supplementary Movie S1). The four different types of targets each had two components: an edible, dead, mealworm, *Tenebrio molitor*, covered by a real or artificial jewel beetle elytron. Iridescent targets were natural wing cases, where iridescence is structurally produced by a chitin multilayer stack with a refractive index contrast phase, probably melanin (Seago et al., 2009), and noniridescent targets were calibrated photographs of the wing cases (Fig. 1a, also see Supplementary Movie S1 for a demonstration of the angle-dependent colour changeability of the iridescent wing cases). The latter displayed the range of colours seen in iridescent elytra, but without any angular change, hereafter referred to as the ‘static spectrum’ treatment. To produce these, we placed a random selection of jewel beetle elytra (*N* = 100) inside the experimental room and photographed them in plan view using a Nikon D90 DSLR camera (Nikon Inc., Tokyo, Japan). Photographs were taken at 1:1 reproduction, and the printed size matched that of the real beetles. Photographs contained an X-Rite ColorChecker Passport (X-Rite Inc., Grand Rapids, MI, U.S.A.), which was used to calibrate the images for bird vision (see e.g. J. Barnett & Cuthill, 2014). The photographs were then printed on glossy photo paper (Epson premium glossy photo paper inkjet S042155) using an Epson SureColor SC-P600 printer with the ‘premium gloss’ setting (Seiko Epson Corporation, Suwa, Japan).

**Figure 2:**
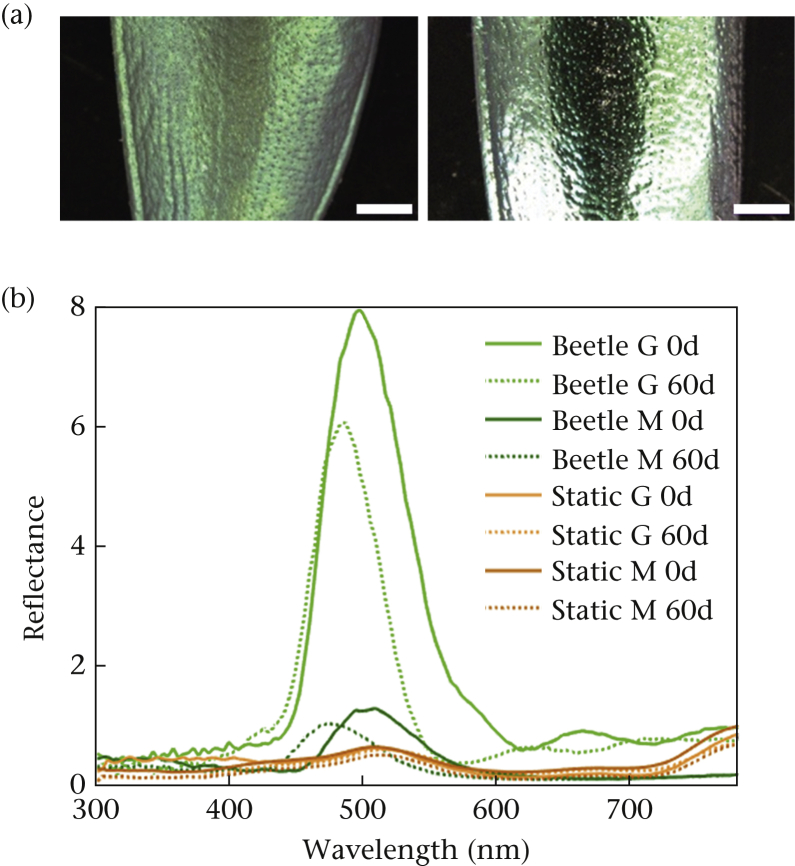
(a) Microscopy images to illustrate the effects of the matte and glossy varnish on the jewel beetle elytra. Scalebar denotes 2 mm. (b) Reflectance spectra measured from one high-intensity-coloured location away from the specular gloss spot on each manipulation of the real jewel beetle and static spectrum targets. G: glossy; M: matte. 0d and 60d indicate 0 degrees and 60 degrees rotation of the beetle in the optical axis. Solid versus dotted lines show an angle-dependent change for reflectance from iridescent targets (green lines), but no angle-dependent change for the static spectrum targets (orange lines).

To manipulate the level of gloss, we sprayed half of the iridescent and half of the static spectrum targets with glossy varnish (Winsor & Newton Professional Gloss Varnish Spray; W & N, London, U.K.), and the other half with matte spray (Winsor & Newton General Purpose Matt Varnish). We quantified the differences in specular reflection between treatments with a ZGM1120 Glossmeter (Zehntner Testing Instruments, Sissach, Switzerland, using software ‘GlossTools’ v.2.1). The Glossmeter provides the ratio of specular to diffuse reflectance (‘gloss units’). We recorded all specular measurements at 60°, because that is the recommended measurement angle for small surfaces (Whitney et al., 2012). The Glossmeter was calibrated using a black polished glass standard supplied with the meter. Five readings were taken from each wing case (*N* = 10 per treatment group), demonstrating the desired difference between the glossy and matte targets (Figure 1, Figure 2, see also Supplementary Movie S1 for an illustration of the ‘glare’ caused by the glossy surfaces in both the iridescent and static spectrum glossy targets, compared to the diffuse scattering of light in both matte target types). Spectral measurements of all four target types were obtained using a hyperspectral camera (sensor: Hamamatsu Orcs 03, Hamamatsu Photonics, Hamamatsu City, Japan; unit: Resonon Pika UV, Resonon, Bozemon, MT, U.S.A.; lens: UV Nikkor 105 mm, Nikon Corp., Tokyo, Japan) with a frequency range of 300–800 nm (Fig. 2). The hyperspectral camera was calibrated using a white PTFE slab (Gilden Photonics Ltd, Glasgow, U.K.). Wide-angle illumination was by four halogen bulbs (STATUS 50W GU10, Status International, Cleckheaton, U.K.) pointing inwards on two perpendicular axes at 45° elevation.

**Figure 3:**
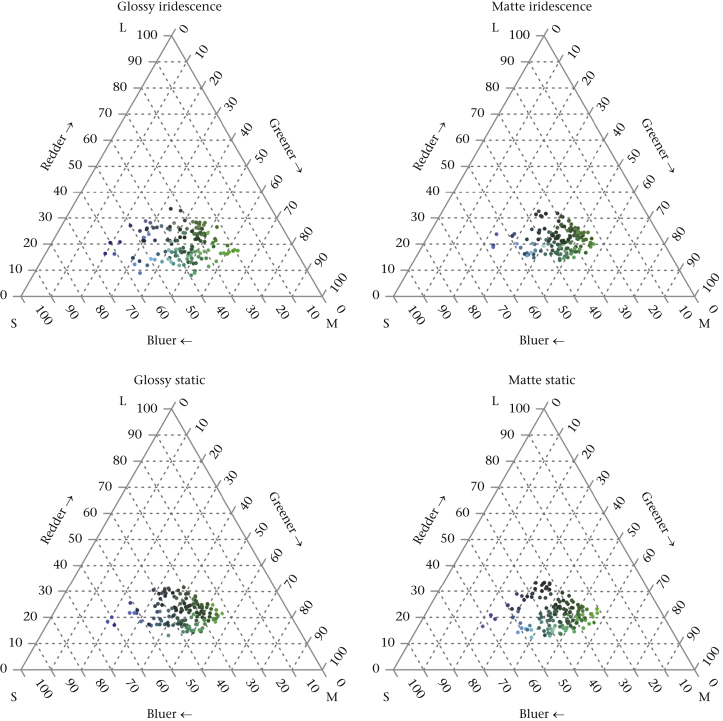
Ternary plots (Smith, 2017) of the colours of the four treatments in an avian chromatic space. Points are 10 sampled pixels from photos of each of 14 targets of each treatment, using a calibrated Nikon D3200 camera (Nikon Corp., Tokyo, Japan). These were transformed to the photon captures of the shortwave (S), mediumwave (M) and longwave (L) cones of a blue tit, *Cyanistes caeruleus*, using the methods in Kjernsmo et al. (2020). The axes of the plots, also known as Maxwell triangles, represent the percentage of photon catches by each cone type. The axes run from the respective apex (100%) to the mid-point of the side opposite (0%) and the centre of the triangle is the achromatic locus (black-grey-white). For illustrative purposes, points are coloured according to human perception.

Note that the colour spectra illustrated in Fig. 2 (and measurements from photographs in Fig. 3) were deliberately taken at points away from the specular reflectance ‘gloss-spot’, and therefore are only a product of the structural colour mechanism generating hue changes. The structural colour green is incredibly bright and strongly directional and, when a matte surface is added on top, much of the brightly reflected light is scattered at other angles, reducing the brightness (but enhancing the consistency of hue across multiple angles). The printed surface of the static spectrum targets, on the other hand, reflects by scattering light in all directions from each dye molecule. The reflectance is not directional, which is why the effect produced by an additional matte coating does not have a noticeable effect on the reflectance intensity here (Fig. 2). The significant, visible and quantifiable difference in the level of specular reflection (glossiness) between the matte and glossy targets for both the iridescent and the noniridescent (static) targets was quantified using a glossmeter (as described above), which measures specular reflectance, and these results are shown in Fig. 1. That the colours away from the gloss spot are very similar across treatments can be seen in the avian colour space representations in Fig. 3.

### Birds and Holding Conditions

We used 51 domestic chicks from Hy-Line Millennium Hatchery (Lower Skilts Farm, Studley, U.K.). The chicks arrived within 24 h of hatching. Once they were settled in their home pens and were eating well, each chick was marked with Porcimark spray (KRUUSE, Langeskov, Denmark) for identification. Of the 51 chicks, 32 were marked as experimental chicks and 19 as ‘buddies’ (the latter served to keep the experimental chicks company in the experimental arenas, following Skelhorn & Rowe, 2006). The chicks were housed in home pens measuring 3 × 1 m with ambient temperature maintained at 25–28 °C and a 12:12 h light:dark cycle using daylight-mimicking lamps (GEWISS, www.gewiss.com; twin 26 W LED). Water and chick starter crumbs (Farmgate, www.forfarmers.co.uk/poultry) were provided ad libitum*,* except during the experimental phase when chicks had a 30 min pretrial deprivation to increase their foraging motivation. Chicks also received mealworms in their home pens, as these were used as rewards in the experiment.

### Ethical Note

The experiment was carried out with the approval of the Animal Welfare and Ethical Review Body, University of Bristol (UIN: UB/17/047). Utmost care was taken to minimize stress, including the use of ‘buddy chicks’ to prevent experimental chicks feeling lonely in the arena, pre-exposure to the foraging board and appropriate ambient lighting. At the end of the experiment and with veterinary approval, all chicks were successfully rehomed to local small-holders.

### Procedure

Chicks were initially trained to eat chick starter crumbs from a foraging plate made from A4-sized cardboard, placed in their home pen, to establish an association between the plate and food. The experiment took place in an identical room opposite their home pens, where there were two identical arenas measuring 120 × 50 cm and 50 cm high. Both arenas contained a section measuring 20 × 50 cm and 50 cm high, partitioned with wire mesh to create a separate ‘buddy area’.

The experiment started on the second day after the arrival of the chicks. Just before the experiment, two chicks were placed in the buddy arena to reduce any potential distress from isolation. These buddy chicks were changed every three trials (or earlier if they started to alarm-call: < 5% of trials).

**Figure 4:**
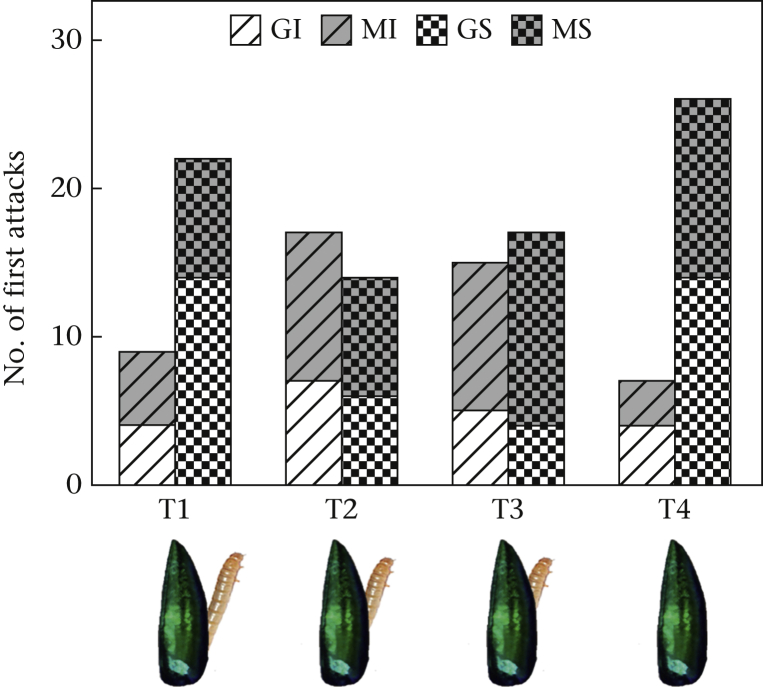
Number of first attacks the chicks directed towards the iridescent prey (I), static spectrum (S), glossy (G) and matte (M) targets for all four trials (T1–T4). Below the graph is an illustration of how the mealworm was gradually hidden under the targets for each trial until it was completely hidden in T4.

Each of the 32 experimental chicks underwent four experimental trials, one per day. In each trial, chicks were presented with all four prey items randomly distributed on the foraging plate. From approaching the foraging plate within pecking distance from the first target, each experimental chick had a maximum of 4 min to attack all four items (i.e. to flip over the artificial prey item to receive the food reward) from the foraging plates (see Supplementary Movie S2 for an example of an experimental trial, including what an ‘aversion response’ looks like). To ensure that the chicks were motivated to attack, during trails 1–3, the mealworm was gradually hidden until it was completely covered by the artificial prey items in trial 4 (Fig. 4). Any targets that were damaged during the experiment were replaced with fresh targets. The order of attack was scored in real time by two observers. However, using ‘order of attack’ as a measure of attack willingness can be challenging, because it can also be argued that the primary data presented here mainly allow conclusions about the relative preference for, rather than avoidance of, a certain prey type. Therefore, direct observations of avoidance behaviour (defined as walking off the foraging plate or actively avoiding a prey item rather than attacking it before the trial ended) was also determined from video recordings obtained from trial 4 (i.e. when the food reward was covered).

### Statistical Analysis

One chick failed to attack any prey in trials 1 and 2; these were treated as missing values. Four chicks attacked some, but not all, targets in trial 1; these prey were assigned a tied rank of 4, equivalent to ‘last to be eaten’.

We analysed attack order using mixed model ordinal logistic regression (function clmm in the package ordinal, Christensen, 2019, pp. 4–25) in R 3.6.1 (R Core Team, 2019). Gloss, iridescence, trial and their interactions were treated as fixed effects, and chick ID as a random effect. Subsequent dissection of where in the attack sequence differences occurred was performed using generalized linear mixed-effect models (GLMM) with binomial error and logit link function (glmer in package lme4, Bates et al., 2015). Significance was tested using likelihood ratio tests, comparing models with and without the factor in question, with stepwise removal of nonsignificant terms, starting with the highest order interaction.

## Results

The order of attack varied significantly with respect to the two-way trial∗gloss and trial∗iridescence interactions (Fig. 2; three-way interaction: χ^2^_3_ = 5.34, *P* = 0.155; gloss∗iridescence: χ^2^_1_ = 0.06, *P* = 0.807; gloss∗trial: χ^2^_1_ = 14.55, *P* = 0.002, iridescence∗trial: χ^2^_1_ = 12.37, *P* = 0.006). These two-way interactions were only evident in the first prey attacked (GLMMs: first prey: gloss∗trial: χ^2^_1_ = 10.27, *P* = 0.016; iridescence∗trial: χ^2^_1_ = 12.49, *P* = 0.006; second prey: gloss∗trial: χ^2^_1_ = 0.999, *P* = 0.802; iridescence∗trial: χ^2^_1_ = 0.928, *P* = 0.819; third prey: gloss∗trial: χ^2^_1_ = 2.56, *P* = 0.464; iridescence∗trial: χ^2^_1_ = 2.16, *P* = 0.541; fourth prey: gloss∗trial: χ^2^_1_ = 6.89, *P* = 0.075; iridescence∗trial: χ^2^_1_ = 6.15, *P* = 0.105).

To investigate the trial-to-trial differences in the main effects of iridescence and gloss, we fitted separate GLMMs to each trial's data. In trial 1, the probability of attacking the first prey did not vary with gloss (matte:gloss odds ratio 0.63, 95% confidence interval, CI 0.26–1.46; χ^2^_1_ = 1.15, *P* = 0.284), but noniridescent prey were 3.27 times (CI 1.39–8.26) more likely to be attacked than iridescent prey (χ^2^_1_ = 7.51, *P* = 0.006). In trial 2, there was no significant effect of gloss (odds ratio 1.54, CI 0.68–3.57; χ^2^_1_ = 1.08, *P* = 0.298) or iridescence (odds ratio 0.77, CI 0.34–1.74; χ^2^_1_ = 0.39, *P* = 0.532). In trial 3, matte targets were 3.43 times more likely to be attacked first than glossy targets (odds ratio 3.43, CI 1.48–8.57; χ^2^_1_ = 8.40, *P* = 0.004), but there was no effect of iridescence (odds ratio 1.20, CI 0.52–2.76; χ^2^_1_ = 0.18, *P* = 0.673). In trial 4, noniridescent prey were 5.27 times (CI 2.16–14.36) more likely to be attacked than iridescent prey (χ^2^_1_ = 14.22, *P* < 0.001) but there was no effect of gloss (odds ratio 0.68, CI 0.29–1.61; χ^2^_1_ = 0.75, *P* = 0.387). Finally, direct observations of the chicks' avoidance behaviour obtained from video recordings in trial 4 revealed that 9/32 chicks walked off the board or actively avoided the prey item before the trial ended (i.e. before all four prey items were attacked). These displays of avoidance behaviour occurred significantly more often when there was only iridescent prey left (eight of nine cases), compared to when there was only static spectrum prey left (one of nine cases; binomial test: *N* = 9, *P* = 0.039).

## Discussion

This experiment demonstrates a possible antipredator function of iridescence, and potentially gloss, against avian predators. Considering that all birds in our experiment were naïve, any behavioural response they showed in their first prey encounter can be considered unlearnt. Interestingly, the birds initially hesitated to attack the iridescent, but not the static spectrum prey: having multiple colours displayed at the same time is not enough to elicit aversion. Rather, it is the key feature of iridescence, its colour changeability, that is important for this protective effect (Kjernsmo et al., 2020). Our study is the first to isolate the effects of gloss from iridescence: a benefit of glossiness over matte appeared in the third trial, consistent with Waldron et al.’s (2017) findings, but iridescence had benefits on both the first and last trial.

The variation in attack willingness across trials could be due to opposing forces: the aversive properties of the coloration and the positive value of the mealworm reward, as with aposematic prey (C. A. Barnett et al., 2012; Skelhorn et al., 2016). We suggest that the aversion to iridescence, while high in trial 1, diminished on subsequent trials and so was balanced by the attraction of the visible mealworm in trials 2 and 3. However, when the reward was completely covered in trial 4, the aversion effect of iridescence dominated once more. How long the aversion effect might persist requires further investigation.

As our prey did not have any secondary defences and yet still induced an aversion response, it is possible that iridescence could facilitate the evolution of aposematism. Future studies could establish the frequency with which the combination of iridescence and secondary defences occurs. As the intensity of the iridescent effect is also dependent on the illuminant, future experiments could investigate the protective function of iridescence and gloss relative to the specularity of the background and the level of, and variation in, ambient light, both of which affect detectability (Cuthill et al., 2019). Nevertheless, our finding that iridescence and, to a lesser extent, gloss induce an aversion response in avian predators further adds to our understanding of the evolution of this enigmatic form of structural coloration. Ultimately, this could provide one explanation for why iridescence is so taxonomically widespread, at least in sexually monochromatic species.

## Author Contributions

K.K., A.L., I.C.C., H.M.W. and N.E.S.-S. conceived the experiment. K.K., A.L., R.M., L.C. and J.R.H. collected the data. K.K. and R.M. conducted spectral measurements of the targets. K.K. and I.C.C. performed the statistical analyses. K.K. wrote the first draft, with contributions by all authors.

## Data Availability

The data are deposited in the University of Bristol data repository: https://doi.org/10.5523/bris.32jo661m7sdkw2751vtphaq7vl.

## Declaration Of Interest

The authors declare no conflict of interest.

## Supplementary material

Supplementary Movie S1 Sample of the four different experimental stimuli used. Supplementary Movie S1

Supplementary Movie S2 Example of an experimental trial. Supplementary Movie S2
